# Multiplex PCR for 17 Y-Chromosome Specific Short Tandem Repeats (STR) to Enhance the Reliability of Fetal Sex Determination in Maternal Plasma

**DOI:** 10.3390/ijms13055972

**Published:** 2012-05-16

**Authors:** Yuan Rong, Jiajia Gao, Xinqiang Jiang, Fang Zheng

**Affiliations:** 1 Center for Gene Diagnosis, Zhongnan Hospital of Wuhan University, Donghu Rd 169, Wuchang District, Wuhan 430071, China; E-Mails: whushine@163.com (Y.R.); jia1104ab@163.com (J.G.); 2 Institute of Forensic Science, Zhongnan Hospital of Wuhan University, Donghu Rd 169, Wuchang District, Wuhan 430071, China; E-Mail: mephiston@163.com

**Keywords:** cell-free fetal DNA, noninvasive prenatal diagnosis, gender determination, Y-chromosome specific STR

## Abstract

The aim of the study was to demonstrate the influence of target gene and amplification product length on the performance of fetal gender determination systems using maternal plasma. A total of 40 pairs of plasma DNA samples from pregnant women and genomic DNA samples from maternal blood, amniotic fluid and paternal blood were isolated for gender determination by amplification of the amelogenin gene and 17 Y-chromosome STR loci, using three different commercial kits. The gender of the fetuses was confirmed by cytogenetic analysis or phenotype at birth. Both the AmpFℓSTR-Identifiler amplification kit and the Mini-STR Amplification kit for amelogenin gene detection were reliable in determining fetal gender (92.0% and 96.0%, respectively), but false negatives were present in both systems. AmpFℓSTR-Yfiler was found to be fully reliable as it amplified Y-STR in all cases of pregnancies with male fetuses and thus was 100% correct in determining fetal gender. The results demonstrated that multiple fluorescent PCR for 17 Y-STR loci was more reliable than *AMELY* gene testing in fetal sex determination with maternal plasma. We also found that the shorter amplification products could improve the performance of fetal gender determination systems.

## 1. Introduction

Prenatal gender determination is used for women at high risk of serious sex-linked genetic disorders. Traditionally, this is undertaken by invasive testing such as chorionic villus sampling or amniocentesis, both of which carry a small but significant (less than 1%) risk of miscarriage and may be harmful for both mother and fetus. The identification of cell-free fetal DNA in 1997 by Dennis Lo in the maternal circulation has allowed the development of non-invasive prenatal diagnostic testing [1], which permits fetal sex determination without risk to the pregnancy.

The identification of Y-chromosome specific DNA sequences in maternal plasma is indicative of a male fetus. To date, many different Y-chromosome specific DNA sequences have been amplified and used to identify male fetuses, including *SRY*, *DYS14*, *DYS1/DAZ*, *DYZ3*, *AMELY*, *etc*. Although results are encouraging, diagnostic accuracy varies according to different methods and target genes used, with sensitivity and specificity ranging from 90% to 100% [2]. The different techniques used for fetal sex determination, such as conventional PCR, nested PCR and real-time PCR, always affect the detection performance. False-negative results are the major drawback with noninvasive prenatal fetal sex determination, simply because there is not enough fetal DNA in the maternal plasma for a 100% successful amplification. To overcome this problem, we adopted a multiple fluorescent PCR technique to detect *AMELY* and 17 Y-STR loci for fetal gender determination.

The amelogenin gene is a chromosome X/Y homologous gene. There is a 6 bp difference in intron 3 of the amelogenin gene between the X-linked amelogenin *(AMELX)* and the Y-linked amelogenin *(AMELY)*. Detection of the 6 bp difference may help us evaluate prenatal fetal gender, which has been widely used in forensic studies [3]. In our study, we used two different amplification kits to amplify *AMELY* to detect fetal gender; we also used multiple fluorescent PCR to observe the influence of the product size of the target gene on the detection performance.

Y-STR are short tandem repeats (STR) that occur specifically on the Y-chromosome, and are therefore unique to the male fetus. The use of Y-STR in forensic evidence allows for the genetic identification of the male component in a mixture of male and female DNA components. Further, even if the amount of female DNA incorporated into the PCR reaction is 100 times more than the amount of male DNA, the female DNA will not interfere with the interpretation of the Y-STR profile [4]. Because of this, the maternal plasma mixture of low copy fetal DNA and high background maternal DNA allows Y-STR genotyping of fetal DNA without interference by maternal background DNA. Therefore, Y-STR typing has the potential to become a useful tool in sex determination using maternal plasma.

In our study, two different amplification systems, the *AMELY* gene test and 17 multiple Y-STR test, were used to determine fetal gender. In the *AMELY* gene test system, we used two kits for amplification of *AMELY* products of two lengths—106–112 bp for the AmpFℓSTR-Identifiler kit and 77–83 bp for the Mini-STR kit—to demonstrate the influence of amplification product length on the performance of fetal gender detection with maternal plasma.

## 2. Results and Discussions

### 2.1. Fetal Gender Confirmation

The gender of fetuses was confirmed by cytogenetic analysis of amniotic fluid or phenotype at birth. In the 40 samples collected, 25 pregnant women had a male fetus.

### 2.2. Fetal Gender Determination by *AMELY* Tests

**Figure 1:**
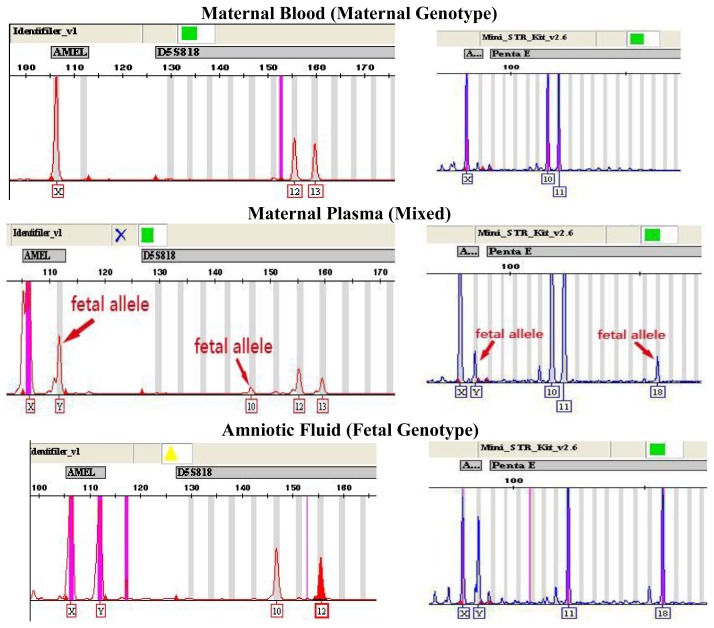
Genotype of *AMELY* and autosomal STR from maternal plasma with a male fetus using the AmpFℓSTR-Identifiler kit (left) and AGCU Mini-STR Amplification kit (right) when compared with maternal blood and amniotic fluid. A third, smaller “paternal” allele is indicated by the arrow, corresponding to the fetal allele that is inherited from the father.

Using the AmpFℓSTR-Identifiler Kit, we were able to successfully amplify Y-chromosome specific *AMELY* in 23 out of 25 maternal plasma samples from mothers bearing male fetuses. Using the Mini-STR amplification kit, *AMELY* was successfully amplified in 24 out of 25 maternal plasma samples with male fetuses (as shown in Table 1). There were no cases of falsely amplified *AMELY* when both the AmpFℓSTR-Identifiler kit and Mini-STR kit were used on samples from mothers bearing female fetuses. When the genotyping results obtained from maternal plasma, maternal blood-cell portion and amniotic fluid were compared, *AMELY* was the only locus that was reliably amplified; autosomal fetal STR loci originating from the father (as shown in Figure 1) were amplified only sporadically due to the allelic suppression.

### 2.3. Fetal Gender Determination by Y-STR Tests Using AmpFℓSTR-Yfiler Kit

On the basis of the presence of detected Y-STR alleles and the value of allelic peak height, AmpFℓSTR-Yfiler Kit was found to be fully reliable as it amplified Y-STR in all 25 cases of maternal plasma from pregnant women with male fetuses, while none of these 17 Y-STR loci was detected in 15 plasma samples from pregnant women carrying female fetuses, and was thus 100% correct in determining fetal gender. All 25 cases of male fetuses were successfully amplified between five and twelve fetal loci (Table 1, Figure 1). An average of 7.8 Y-STR loci was detected. In addition, the genotype of all the amplified Y-STR derived from the maternal plasma matched the alleles amplified from the corresponding amniotic fluid and paternal blood, thus further supporting the fact that the amplified fragments originated from the Y-chromosome, and not from nonspecific amplification of another DNA fragment (as shown in Table 2).

### 2.4. Discussion

In the noninvasive prenatal fetal gender determination from maternal plasma, there are several factors which will affect the detection performance, including sample handling and DNA extraction efficiency, amplification techniques and the gestational age [2].

In this study, we demonstrated the effect of both amplification product size and the different target genes on fetal gender determination. The principle of the commercially available forensic sex tests is based on the polymorphism present on the two homologous copies of the amelogenin gene on the X and Y chromosomes *(AMELX/AMELY).* The primer pairs used for genotyping are those described by Sullivan *et al*., detecting a 6 bp deletion/insertion on the X/Y chromosomes [5]. The relatively short amplicon length of 106 and 112 bp, respectively, is usually sufficient for routine DNA typing. However, with highly degraded DNA, even this stretch might be too long for adequate analysis. Fetal DNA molecules in the maternal plasma are generally shorter than maternal DNA molecules, less than 150 bp in length [6]. On the basis of the characteristics of circulatory fetal DNA in maternal plasma, we used a Mini-STR amplification kit in which all amplified allele sizes were less than 250 bp in length, to detect the *AMEL* gene with even shorter amplicons of 77–83 bp, to study the influence of the allele size on the detection performance. According to the results of our study, both the AmpFℓSTR-Identifiler amplification kit and Mini-STR Amplification kit were reliable in amplifying the *AMELY* gene to determine fetal gender (92.0% and 96.0%, respectively). There was no statistically significant difference between the two kits. The single difference of incorrect samples between them may be a result of the biochemical character of circulatory fetal DNA. It was showed that the STR loci with shorter allele size had a greater chance of being amplified in both the fetal autosomal STR detecting system and the Y-STR detecting system than the STR loci with longer allele size. The autosomal STR loci were amplified only sporadically, most probably because of suppressed primer binding due to the presence of high-background maternal DNA. For the genotyping of Y-STR from maternal plasma, the STR loci with shorter allele sizes, such as DYS456, with an allele size ranging from 100–127 bp, and DYS393 (106–144 bp), were amplified in 23 and 20 out of 25 maternal plasma samples with male fetus, respectively, while the STR loci with longer allele sizes, such as DYS392 (286–335 bp) and DYS635 (242–274 bp), were only amplified in 2 and 1 out of 25 maternal plasma samples with male fetus, respectively.

Although fetal sex determination with both systems of detecting the *AMELY* gene was reliable, false-negative results were still present. Suppression of minor alleles is a common complication in the analysis of low-copy DNA samples. It has been reported that, on average, 3–6% of cell-free DNA in maternal plasma can be of fetal origin [7]. A higher fraction (around 10–20%) of fetal DNA has been found in maternal plasma using microfluidics digital PCR [8].

The absence of amplification of *AMELY* in these cases could have been a consequence of inadequate quantity of fetal DNA and the corresponding allelic suppression, but this was probably not the only reason, since different methods gave false negatives in different individuals. Individual false negative samples are listed in Table 1. Even some samples with good results by Y-STR amplification were falsely negative by *AMELY* detection, which strongly supports the hypothesis that falsely negative results may be caused by some type of variability in primer binding regions; inadequate amplification efficiency of different primers or the Y-chromosome micro-deletion were actually not very common but existed in some cases.

**Figure 2:**
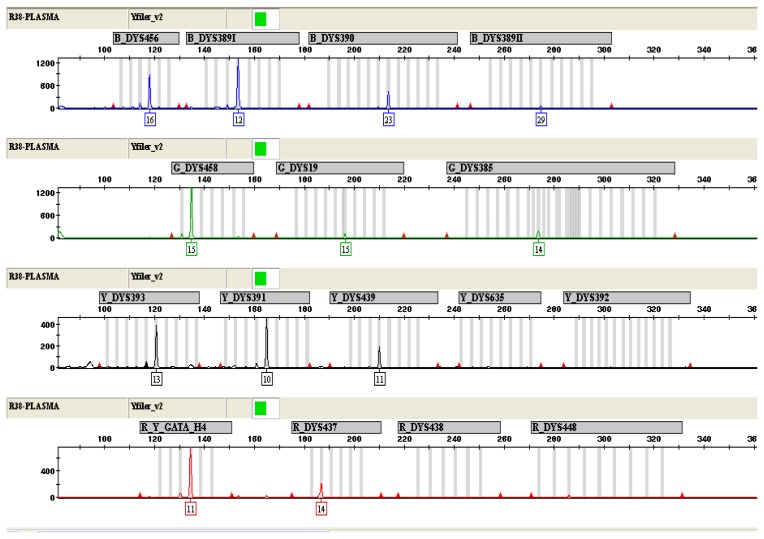
Y-STR genotyping of DNA from maternal plasma with a male fetus (16 weeks of pregnancy). 12 out of 17 STR loci were successfully amplified.

To eliminate these problems mentioned above, the AmpFℓSTR-Yfiler system was used to amplify 17 STR loci located on the Y-chromosome in parallel. Since primers from this system do not bind to maternal background DNA, the allelic suppression could be avoided and significantly better results were obtained. Between five and twelve fetal loci were successfully amplified from plasma of mothers bearing male fetuses (Table 1, Figure 2). All amplified alleles matched to alleles detected in samples of paternal blood, excluding the possibility of falsely positive results caused by DNA contamination. Furthermore, the method that the 17 Y-STR multiplex amplification kit utilizes in fetal gender determination can also avoid the false-negatives resulting from individual variability in primer binding region and Y-chromosome micro-deletion. Although these problems do not occur frequently, they may be the reason for the false-negative results when using the single Y-chromosome specific gene detection for fetal gender determination such as the *SRY* or *AMELY* gene [9].

The multiple Y-STR loci amplification can overcome this problem by amplifying two or more Y-chromosome specific target regions in parallel, which can be complementary to each other and improve the detection accuracy. In addition, Y-STR testing may be useful in some cases of fetal sex chromosome abnormality. The male fetuses with Supermale Syndrome (XYY) may cause the appearance of two peaks at one or more Y-STR loci while other sex chromosome aneuploidy diseases such as XXY, XO, XXX and mosaic conditions cannot be identified by Y-STR testing because fetal sex chromosome dosage cannot be accurately evaluated with maternal plasma by this method.

Our results need to be confirmed by further studies in a larger clinical cohort and at an earlier gestational age because larger clinical trials reporting the sensitivity and specificity of this diagnostic procedure are unavailable. Moreover, as an ethical issue, this method might also be misapplied in prenatal sex selection. Therefore, there should be careful consideration about the use of this analytical tool in clinical situations. Note should be taken that fetal sex determination for non-medical reasons is unethical and prohibited in some countries.

## 3. Experimental Section

### 3.1. Participant Recruitment and Sample Collection

This study was performed in accordance with the Declaration of Helsinki and approved by the local ethical committee of Zhongnan Hospital of Wuhan University. Peripheral blood samples (*n* = 40) were collected from pregnant women who had a gestational age of 12–36 weeks. The corresponding amniotic fluid and blood samples from fathers were also collected. Informed consent was obtained from all studied individuals.

### 3.2. Sample Processing and DNA Extraction

Maternal peripheral blood samples (4 mL) were drawn and collected in tubes containing EDTA as anticoagulant, and the blood samples were stored at 4 °C before centrifugation. The blood centrifugation was performed within 6 h after collection. The blood samples were separated into plasma and blood cells by centrifuging at 1600× *g* for 10 min at 4 °C. The plasma portion and the blood-cell portion were re-centrifuged at 16,000× *g* for 10 min at 4 °C to remove any residual blood cells or plasma and were stored at −80 °C before further processing.

Cell-free DNA was extracted from 2.0 mL of plasma with the QIAamp Ultrasens Virus Kit (Qiagen, Germany) according to the manufacturer’s protocol.

DNA from the maternal blood-cell portion, paternal peripheral blood and amniotic fluid were extracted by chelex-100 method [10].

### 3.3. Amplification Using Applied Biosystems AmpFℓSTR-Identifiler Kit

DNA from maternal plasma, maternal blood-cell portion and amniotic fluid were amplified using AmpFℓSTR-Identifiler Kit (Applied Biosystems, Foster City, CA). PCR reaction was performed in a final volume of 10 μL containing 4 μL AmpFℓSTR PCR reaction mix, 2 μL AmpFℓSTR-Identifiler primer set, 0.33 μL AmpliTaq Gold DNA Polymerase(5 U/μL) and 3.67 μL template DNA. The thermal profile was 95 °C for 11 min, followed by 28 cycles of 94 °C for 60 s, 59 °C for 60 s, 72 °C for 60 s, and 60 °C for 45 min. The AmpFℓSTR-Identifiler Kit amplifies 15 autosomal STR loci plus the amelogenin gene. These loci include: CSF1PO, D13S317, D16S539, D18S51, D21S11, D3S1358, D5S818, D7S820, D8S1179, FGA, TH01, TPOX, vWA, D2S1338 and D19S433.

### 3.4. Amplification Using AGCU Mini-STR Amplification Kit

DNA from maternal plasma, maternal blood-cell portion and amniotic fluid were amplified using Mini-STR Amplification kit (AGCU, China). PCR reaction was performed in a final volume of 10 μL containing 4 μL AGCU PCR Reaction Mix, 2 μL Mini Primer Set, 0.2 μL Hot-start G-taq DNA Polymerase (5 U/μL) and 3.8 μL template DNA. The thermal profile was 95 °C for 11 min, 10 cycles of 94 °C for 60 s, 62 °C for 60 s, 72 °C for 60 s, followed by 20 cycles of 90 °C for 60 s, 60 °C for 60 s, 72 °C for 60 s, and 60 °C for 45 min. The Mini-STR Amplification Kit amplifies 8 autosomal STR loci and the amelogenin gene. These loci include: Penta E, D21S391, D6S1043, D2S1338, CSF1PO, Penta D, D19S253.

### 3.5. Amplification Using AmpFℓSTR-Yfiler Kit

DNA from maternal plasma, paternal blood-cell and amniotic fluid were amplified using AmpFℓSTR-Yfiler Kit (Applied Biosystems, Foster City, CA, USA). PCR reaction was performed in a final volume of 10 μL containing 4 μL AmpFℓSTR PCR Reaction Mix, 2 μL AmpFℓSTR-Yfiler Primer Set, 0.33 μL AmpliTaq Gold DNA Polymerase(5 U/μL) and 3.67 μL template DNA. The thermal profile was 95 °C for 11 min, followed by 30 cycles of 94 °C for 60 s, 61 °C for 60 s, 72 °C for 60 s, and 60 °C for 80 min. The AmpFℓSTR-Yfiler PCR Amplification Kit amplifies 17 loci: DYS456, DYS389I, DYS390, DYS389II, DYS458, DYS19, DYS385a/b, DYS393, DYS391, DYS439, DYS635, DYS392, Y_GATA_H4, DYS437, DYS438, and DYS448.

### 3.6. Analysis of Amplified Products

These amplified products were separated and detected by capillary electrophoresis on the ABI 3130 Genetic Analyzer using the GeneMapper ID v3.2 software for analysis with the corresponding analysis method.

## 4. Conclusion

In summary, the shorter length of amplification products could improve the performance of fetal gender determination. Multiple fluorescent PCR for 17 Y-chromosome STR loci was more reliable in prenatal sex determination than *AMELY* testing, which could both enable the comparison of Y-STR genotype between father and child to avoid the false positive results, and also amplify multiple Y-STR loci simultaneously to avoid the false negative results.

## Figures and Tables

**Table 1:** Fetal gender determination by maternal plasma samples with male fetuses using two different target regions (*AMELY* test and Y-STR detection).

Sample No.	Gestational age (weeks)	*AMELY* Test		Multiple Y-STR Amplification	

		AmpFℓSTR Identifiler Kit	AGCU Mini-STR Kit	Amplified Y-STR Loci	Matching to Paternal Blood
1	15	XY	XY	5	5
2	16	XY	XY	7	7
3	24	XY	XY	11	11
4	28	**XX**	XY	6	6
5	26	XY	XY	9	9
6	28	XY	XY	10	10
7	27	XY	XY	10	10
8	36	XY	XY	8	8
9	35	XY	XY	8	8
10	28	XY	XY	10	10
11	16	**XX**	XY	7	7
12	36	XY	XY	10	10
13	23	XY	XY	8	8
14	15	XY	**XX**	6	6
15	16	XY	XY	12	12
16	12	XY	XY	5	5
17	13	XY	XY	6	6
18	17	XY	XY	8	8
19	24	XY	XY	5	5
20	14	XY	XY	6	6
21	22	XY	XY	8	8
22	20	XY	XY	8	8
23	18	XY	XY	7	7
24	22	XY	XY	9	9
25	15	XY	XY	7	7

**Table 2:** Comparison of polymorphic Y-chromosome STR loci between father and fetus. The genotype of a fetus from maternal plasma was shown in a tabular form and compared to the genotype obtained from paternal blood. All Y-STR alleles amplified from maternal plasma were identical to paternal alleles, which further confirmed that they indeed originated from the fetal Y chromosome.

	DYS456	DYS389 I	DYS390	DYS389 II	DYS458	DYS19	DYS385a/b	DYS393
maternal plasma	16	12	23	29	15	15	14,14	13
paternal blood	16	12	23	29	15	15	14,14	13

	**DYS391**	**DYS439**	**DYS635**	**DYS392**	**Y_GATA_H4**	**DYS437**	**DYS438**	**DYS448**

maternal plasma	10	11	20	14	11	14	10	18
paternal blood	10	11	-	-	11	14	-	-
